# Current status and proposed roles for nitric oxide as a key mediator of the effects of extracellular nucleotides on plant growth

**DOI:** 10.3389/fpls.2013.00427

**Published:** 2013-10-25

**Authors:** Mari L. Salmi, Greg Clark, Stanley J. Roux

**Affiliations:** Department of Molecular Biosciences, The University of Texas at AustinAustin, TX, USA

**Keywords:** nitric oxide, extracellular nucleotides, apyrase, auxin transport, post-translational modifications, *S*-nitrosylation, Tyr-nitration, peroxynitrite

## Abstract

Recent data indicate that nucleotides are released into the extracellular matrix during plant cell growth, and that these extracellular nucleotides induce signaling changes that can, in a dose-dependent manner, increase or decrease the cell growth. After activation of a presumed receptor, the earliest signaling change induced by extracellular nucleotides is an increase in the concentration of cytosolic Ca^2+^, but rapidly following this change is an increase in the cellular level of nitric oxide (NO). In *Arabidopsis*, mutants deficient in nitrate reductase activity (*nia1nia2*) have drastically reduced nitric oxide production and cannot transduce the effects of applied nucleotides into growth changes. Both increased levels of extracellular nucleotides and increased NO production inhibit auxin transport and inhibit growth, and these effects are potentially due to disruption of the localization and/or function of auxin transport facilitators. However, because NO- and auxin-induced signaling pathways can intersect at multiple points, there may be diverse ways by which the induction of NO by extracellular ATP could modulate auxin signaling and thus influence growth. This review will discuss these optional mechanisms and suggest possible regulatory routes based on current experimental data and predictive computational analyses.

## INTRODUCTION

For over 40 years scientists have known that the main energy currency of the cell, ATP, is sometimes released by cells into their extracellular matrix (ECM). In the ECM, ATP functions not primarily to drive energy-dependent reactions, but primarily to bind to receptors and activate signaling changes (Khakh and Burnstock, 2009). The data on this topic have come almost exclusively from studies on animals. However, during the last 10 years an increasing number of reports have demonstrated that signaling changes induced by extracellular ATP (eATP) and other nucleoside triphosphates and diphosphates are a common phenomenon also in plants (Tanaka et al., 2010; Clark and Roux, 2011). In animals, these signaling changes are known to begin with the activation of well-characterized receptors, termed purinoceptors, which fall into two main categories: P2X, which are ion-channel linked, and P2Y, which are G-protein linked. In plants, too, there is strong indirect evidence that there may be at least two kinds of plasma-membrane-localized receptors for extracellular nucleotides (Demidchik et al., 2009, 2011), but their primary structures are clearly different from the animal purinoceptors, and as yet their identity is unknown (Clark and Roux, 2011).

The processes that release ATP and other nucleotides into the ECM are similar in plants and animals and include secretion, active transport, and wound or pathogen events that break the plasma membrane or make it leaky (Roux and Steinebrunner, 2007). Animals and plants also use similar enzymes to limit the build-up of eATP, primarily ecto-nucleoside triphosphate diphosphohydrolases (ecto-NTPDases) or ectoapyrases, which remove the terminal phosphate from nucleoside triphosphate diphosphohydrolases (NTPs) and nucleoside diphosphohydrolases (NDPs; Knowles, 2011).

 Although the search to identify plant purinoceptors has not yet yielded definitive results, the signaling changes induced by extracellular nucleotides in animals and plants are already known to be remarkably similar. They begin with a rapid increase in the concentration of free cytosolic calcium ([Ca^2+^]_cyt_; Demidchik et al., 2003, 2009, 2011; Jeter et al., 2004; Burnstock et al., 2010). Afterward, early downstream changes include increased production of superoxide and NO (D’Andrea et al., 2008; Clark and Roux, 2009; Harada, 2010; Tanaka et al., 2010). Sueldo et al. (2010) reported that eATP-induced NO production is downstream of phosphatidic acid production in suspension cultured tomato cells. In plants, mutants that are suppressed in their ability to make either superoxide or nitric oxide are insensitive to the effects of applied nucleotides on cell growth (Clark et al., 2010) and stomatal aperture (Clark et al., 2011; Hao et al., 2012), which suggest these signaling intermediates are needed to convert eATP receptor activation to physiological changes in cells.

This review focuses on the requirement for NO production to transduce extracellular nucleotide signals into growth and other physiological changes in plants. Because there is strong evidence linking extracellular nucleotide effects to changes in auxin transport (Tang et al., 2003; Liu et al., 2012), the question of how eATP-induced changes in NO production could alter auxin transport becomes especially relevant. As noted by Liu et al. (2012), current evidence favors post-transcriptional events as being key to changing the molecular activities that drive auxin transport, so this review will especially highlight the role of NO in protein modifications that could rapidly alter either the transport or concentration of auxin in cells.

## KINETICS OF SIGNALING RESPONSES INDUCED BY EXTRACELLULAR NUCLEOTIDES

 Two of the better-documented occasions of ATP release by plant cells are wounding (Song et al., 2006) and cell expansion (Kim et al., 2006; Wu et al., 2007; Clark et al., 2011). Wounding, of course, breaks the plasma membrane and allows the leakage of cytoplasmic ATP (concentration ∽mM; Gout et al., 1992) into the ECM. Cell expansion is thought to require the delivery of secretory vesicles to the plasma membrane, and, based on the animal literature, these vesicles can carry up to mM ATP and release it into the ECM upon their fusion with the plasma membrane (Lazarowski et al., 2003). Alternatively, plant cells also release ATP when their membranes are stretched or mechanically stimulated (Jeter et al., 2004; Weerasinghe et al., 2009), and membranes are certainly stretched when plant cells expand. Both wounding and cell expansion would release ATP quickly, and once in the ECM, ATP, and ADP can induce calcium transport changes and thus initiate signal transduction in less than 30 s (Demidchik et al., 2009, 2011).

 Downstream of the increase in [Ca^2+^]_cyt_ induced by extracellular nucleotides are increases in the production of both reactive oxygen species (ROS; e.g., superoxide and H_2_O_2_; Kim et al., 2006; Song et al., 2006; Tonon et al., 2010; Sun et al., 2012) and nitric oxide (Foresi et al., 2007; Wu and Wu, 2008; Reichler et al., 2009; Clark et al., 2010, 2011). Mutant analyses indicate that both of these changes are needed for the growth and other cellular changes induced by extracellular nucleotides (Clark et al., 2010, 2011). Mutants null for the D/F subunit of NADPH oxidase, which catalyzes superoxide production, or *nia1nia2* double knockouts, which are null for two genes encoding the nitrate reductase enzyme that accounts for significant fraction of the NO production in root hairs (Clark et al., 2010), do not show growth or stomatal aperture responses to applied nucleotides (Reichler et al., 2009; Clark et al., 2010, 2011). Two recently described signaling mechanisms that might link the eATP-induced increase in [Ca^2+^]_cyt_ and the activation of NADPH oxidase activity should be evaluated; a calmodulin-domain protein kinase (CDPK), which can activate NADPH oxidase by phosphorylation (Yoshioka et al., 2011), and the NO-mediated regulation of NADOH oxidase by *S*-nitrosylation (Yoshioka et al., 2011; Yun et al., 2011). The link between increased [Ca^2+^]_cyt_ and activation of nitrate reductase is not as well documented, although this enzyme activity may also be regulated by phosphorylation (Lea et al., 2004). Wu and Wu (2008) found that eATPγS must induce an increase [Ca^2+^]_cyt_ in hairy roots in order to stimulate NO production. Of course, there are other sources of NO production in plants in addition to nitrate reductase, and there is evidence for cross-talk between these enzymes and Ca^2+^ signaling (Besson-Bard et al., 2008).

 Given the rapidity of eATP release by cells and the need for NO production to transduce the eATP signal into cellular changes, it is surprising that until now the earliest detection of NO production is 10 min or more after applied nucleotide treatment (Reichler et al., 2009; Clark et al., 2010). This delayed detection may reflect limitations of the assay methods more than actual delay in NO production. Nonetheless, knowing more precisely the kinetics of nucleotide-induced NO production will be important for determining whether NO plays a primary or secondary role in mediating the broad effects of eATP on plant cell growth and physiology, and specifically on auxin transport.

## NITRIC OXIDE-DEPENDENT PROTEIN MODIFICATIONS

 Two widely studied post-translational modifications (PTMs) that result from an increase in NO are nitration of tyrosine residues generating modified 3-nitrotyrosines, and nitrosylation of cysteine residues (*S*-nitrosylation). Thorough reviews of the proteomic approaches used in plants to identify NO signaling factors (Bykova and Rampitsch, 2013; Jacques et al., 2013; Kovacs and Lindermayr, 2013) and reviews of the various signaling pathways in plants that employ NO-dependent PTM (Astier and Lindermayr, 2012; Corpas et al., 2013) were recently published, so we will not attempt to replicate these. We will focus on what mechanisms link NO-mediated modifications to eATP effects on plant growth generally and auxin transport specifically.

 To understand this regulation it will be important to confidently identify which proteins are *S*-nitrosylated or Tyr-nitrated, and then evaluate if these modifications play a central role in growth control. A standard method for detecting *S*-nitrosylation of proteins, called the biotin-switch method, relies on NO donor treatment of samples prior to identification of modified proteins (Jaffrey et al., 2001). *S*-nitrosylation of *Arabidopsis* proteins was detected by this method after NO-donor treatment in cell suspension culture extracts and leaf tissue (Lindermayr et al., 2005). More recently, a modification of this method that does not rely on application of an NO donor was used to identify endogenously *S*-nitrosylated *Arabidopsis* proteins, again from cell culture (Fares et al., 2011). Detailed analysis of specific plant proteins modified by *S*-nitrosylation demonstrates that this modification can regulate protein activity (Astier and Lindermayr, 2012; Feng et al., 2013).

 The nitrotyrosine PTM has been experimentally detected in proteins of only a few plant systems to date, including 2-week-old *Arabidopsis* whole seedlings (Lozano-Juste et al., 2011), hypocotyls of 9-day-old sunflowers (Chaki et al., 2009), and pea plants at several different stages of development (Begara-Morales et al., 2013). Each of these studies has demonstrated a regulatory role for the Tyr-nitration observed in at least one protein, as well as identified numerous other targets for this PTM.

Several *Arabidopsis* proteins that have been experimentally shown to be *S*-nitrosylated or Tyr-nitrated (Lindermayr et al., 2005; Fares et al., 2011; Lozano-Juste et al., 2011) may play a role in eATP signaling pathways because they function in auxin transport or signaling, in ROS signaling, or in wall extensibility. Included in the small number of plant proteins whose regulation by NO has been experimentally validated is the auxin receptor TIR1 (Terrile et al., 2012). This finding indicates that the regulation of growth and development by auxin includes, in at least one case, NO-dependent PTM of a key protein in auxin signaling, and supports the need to evaluate additional players in this signaling pathway as targets for these regulatory modifications.

There is growing evidence for cross-talk between NO and auxin signaling pathways in root growth and morphology and in responses to iron deficiency (Simontacchi et al., 2013). For example, auxin and NO are both implicated in heavy metal stress responses (Peto et al., 2011; Xu et al., 2011; Kolbert et al., 2012) and in the formation of both adventitious and lateral roots (Pagnussat et al., 2003, 2004; Correa-Aragunde et al., 2004; Lanteri et al., 2006, 2008; Guo et al., 2008; Liao et al., 2011; Yadav et al., 2011; Li and Jia, 2013). Increasing NO levels in *Arabidopsis* primary roots results in a decrease of the polar auxin transport mediated by PIN-FORMED 1 (PIN1), and consequent growth inhibition (Fernandez-Marcos et al., 2011). More recently, auxin was suggested to control root morphology by inducing *S*-denitrosylation of an ascorbate peroxidase enzyme involved in redox regulation (Correa-Aragunde et al., 2013). Nitric oxide also plays a role in auxin-induced stomatal opening (She and Song, 2006). Speculatively, this auxin–NO connection could apply also to effects mediated by extracellular nucleotides, as both eATP and apyrase expression influence auxin transport (Tang et al., 2003; Liu et al., 2012), and both regulate stomatal aperture (Clark et al., 2011) and cell growth (Clark et al., 2010) in an NO-dependent manner.

## SPECULATION ON POSSIBLE ROLES FOR NITRIC-OXIDE BASED POST-TRANSLATIONAL REGULATION IN eATP RESPONSES

Unraveling the specific steps of any signaling pathway is a complicated task, because signaling typically occurs in a feedback process wherein well-characterized steps such as increased [Ca^2+^]_cyt_ and protein phosphorylation are the most commonly observed. While studies have shown that both NO-mediated PTMs mentioned above are reversible and can induce physical changes to proteins that regulate their activity (Lindermayr et al., 2005; Lozano-Juste et al., 2011), another interesting possibility that remains to be studied is an indirect regulatory role for these modifications. Bykova and Rampitsch (2013) suggest that these NO-dependent changes might serve to reversibly occupy an amino acid that could be otherwise modified in a way that would lead to a different activity change (i.e., phosphorylation, carbonylation, or disulfide bond formation).

Predictive, sequence-specific models have been developed to identify sites of cysteine–nitrosylation (Xue et al., 2010) and tyrosine nitration (Liu et al., 2011). These algorithms were developed from K-means clustering methods, and can be used with different thresholds of reliability, high/medium/low, based on the false discovery rate (FDR; 10%/15%/20%). Like efforts to experimentally identify the protein modifications directly, these predictive models are new and still being optimized, but they can be used to identify potential targets of study for these regulatory modifications.

Apyrase proteins serve to regulate extracellular ATP concentration in animal cells (Plesner, 1995; Gaddie and Kirley, 2010), and a similar role may exist for these proteins in plant cells. The *Arabidopsis* nucleoside triphosphate–diphosphohydrolases termed apyrase 1 and 2 have been implicated in e-ATP signaling (Clark et al., 2011; Liu et al., 2012), although they may do so from a Golgi locale (Chiu et al., 2012; Schiller et al., 2012) rather than from a plasma membrane site. When ecto-apyrase activity is inhibited by antibodies raised to APY1 and APY2, the [eATP] of media in which pollen tubes are growing rises several fold and pollen tube growth is inhibited (Wu et al., 2007). Similarly, when APY1/APY2 expression is suppressed by RNAi in R2-4A mutants, this raises the [eATP] of the media and inhibits seedling growth (Salmi, Kim and Roux, unpublished). Although the expression/and or activity of APY1 and 2 appear to influence [eATP], and sites of [eATP] release in roots coincide with sites of increased expression of *APY1* and *APY2* (Roux et al., 2008), it is of course possible that the Golgi function of APY1 and APY2 could regulate growth independent of their influence on [eATP]. Theoretically, other members of the apyrase family could also help regulate [eATP]. At least one Tyr-nitration or *S*-nitrosylation site is predicted in the proteins encoded by each of the seven members of the *Arabidopsis thaliana* family of apyrase genes (**Table 1**; Yang et al., 2013), and these predictions should be experimentally evaluated.

**Table 1:** Computationally predicted NO mediated modifications of proteins implicated in eATP signaling (Xue et al., 2010; Liu et al., 2011). Only predictions included in the “high” threshold category are included here (10% FDR).

**Gene name**	**AGI code**	***S*-nitrosylation**		**Tyr-nitration**	
		**Position**	**Peptide**	**Position**	**Peptide**
PLD1, Phospholipase D α 1	AT3G15730	739	LDPSSLE**C**IEKVNRI	77	EPKNPKW**Y**ESFHIYC
				618	LEEDPRN**Y**LTFFCLG
				803	ILGTKSD**Y**LPPILTT
PLD2, Phospholipase D α 2	AT1G52570	211	KNYEPHR**C**WEDIFDA	15	GRLHATI**Y**EVDHLHA
		739	LDPSSQE**C**IQKVNRV	618	EGEDPRD**Y**LTFFCLG
PLC, Phospholipase C	AT5G58670	11	SFKVCFC**C**VRNFKVK	254	STKPPKE**Y**LQTQISK
		226	FGGSLFQ**C**TDETTEC		
GPA1, G-protein α Subunit 1	AT2G26300	5	MGLL**C**SRSRHHT	74	DEGELKS**Y**VPVIHAN
				106	NETDSAK**Y**MLSSESI
AGG1, G-protein γ subunit 1	AT3G63420	54	TDIVSTV**C**EELLSVI	None predicted
AGG2, G-protein γ subunit 2	AT3G22942	56	MDNASAS**C**KEFLDSV	None predicted
Apyrase 1	At3g04080	322	SGASLDE**C**RRVAINA	None predicated
Apyrase 2	At5g18280	None predicted		8	MLNIVGS**Y**PSPAIVT
				410	PLEGEDS**Y**VREMYLK
Apyrase 3	At1g14240	None predicted		473	VVTPNSD**Y**NGKSRKY
				480	YNGKSRK**Y**LGF
Apyrase 4	At1g14230	54	IIFVIVA**C**VTIALGL	11	SGSDEGV**Y**AWVVANH
		342	AAGNFSE**C**RSAAFAM	303	DLSNVAK**Y**KI
Apyrase 5	At1g14250	334	AAGDFTK**C**RSATLAM	None predicted
Apyrase 6	At2g02970	352	AGGNYSQ**C**RSAALTI	40	APSSSST**Y**TLTKPNS
				349	SFQAGGN**Y**SQCRSAA
				549	YDLEKGR**Y**IVTRIR
Apyrase 7	At4g19180	None predicted		67	SLQDFSS**Y**HGFDPEE

*Proteins AGB1 and GBR1 are not included because neither modification was predicated at any residue.*

*The position of the cysteine or tyrosine amino acid predicted to be modified is give and indicated in bold.*

Nitric oxide production is necessary for the cellular response to extracellular nucleotides (Clark et al., 2010, 2011). Similarly, respiratory burst oxidases also play a critical role in mediating plant responses to eATP (Suzuki et al., 2011). Moreover, the timing of e-ATP-induced production of NO and ROS is similar (both within ∽30–45 min; Clark et al., 2010, 2011). To the extent that NO and ROS are induced at about the same time and are both needed for plants to respond to eATP, these results suggest the possibility that peroxynitirite could serve to induce Tyr-nitration and thus serve as an important regulator of its own production in a feedback mechanism.

Peroxynitrite (ONOO^-^) is a potent oxidant and nitrating species that can be formed by the reaction of NO and $$$ when both of these signaling molecules are present at the same time in the same cell. Plant biologists are just beginning to assay the role of peroxynitrite in plant growth and development (Leitner et al., 2009; Arasimowicz-Jelonek and Floryszak-Wieczorek, 2011; Vandelle and Delledonne, 2011), and thus far it is implicated in hypersensitive defense responses (Saito et al., 2006; Leitner et al., 2009; Gaupels et al., 2011; Bellin et al., 2013) and root development and senescence (Gaupels et al., 2011; Begara-Morales et al., 2013). Peroxynitrite can oxidize proteins and membrane lipids causing cellular damage, and its formation is most likely controlled by the local production of superoxide (Vandelle and Delledonne, 2011). Through this peroxynitrite mechanism, oxidation of lipids may play a role in plant lipid signaling pathways (Sanchez-Calvo et al., 2013).

Could peroxynitrite help mediate some eATP effects? Chivasa et al. (2005) have proposed a role for eATP in programmed cell death, and there is evidence that peroxynitrite may help mediate programmed cell death in plant cells (Serrano et al., 2012a, 2012b). eATP has also been implicated in plant pathogen responses (Chivasa et al., 2009), and a signaling role for ONOO^-^ in these responses has been documented (Saito et al., 2006; Gaupels et al., 2011). More interesting is the potential role for ONOO^-^ in plant growth responses to applied eATP. A growth-inhibiting concentration of eATP produces high levels of NO and ROS, while lower, growth-promoting concentrations of eATP induce low levels of NO and ROS (Clark et al., 2010). Both situations may lead to the production of peroxynitrite, which could mediate the growth regulatory effects of eATP.

Another important target for peroxynitrite-mediated nitration in animal cells is the second messenger cyclic guanosine monophosphate (cGMP; Akaike et al., 2010; Sawa et al., 2013). Recently, a role of nitrated cGMP (8-nitro-cGMP) in Abscisic acid (ABA)-induced stomatal closing was discovered (Joudoi et al., 2013). Because ABA-induced stomatal closure can be partially blocked by mammalian purinoceptor antagonists (Clark et al., 2011), and treating *Arabidopsis* leaves with high levels of eATP induces both NO and ROS, it will be important to determine if eATP treatment causes nitration of cGMP in guard cells. Recently, cGMP was shown to promote lateral root formation in *Arabidopsis* by regulating polar auxin transport (Li and Jia, 2013). Thus, a plausible speculation is that nitration of cGMP might also play an important role in regulating auxin transport.

The effects of exogenously applied ATP and ATP analogs are pronounced in root development (Lew and Dearnaley, 2000; Tang et al., 2003; Wu and Wu, 2008; Wu et al., 2008). Proper localization of auxin is necessary for normal root development. In the apyrase mutants described by Liu et al. (2012) localization of several auxin transporters and the abundance of transcripts encoding these transporters were not altered in plants with inhibited auxin transport and stunted and altered root anatomy. One mechanism for this could be regulation of the transporter activity, and NO-mediated PTMs are likely candidates for this regulation. Several proteins known to be involved in polar auxin transport have predicted *S*-nitrosylation and Tyr-nitration sites (**Table 2**), and these predictions should be experimentally evaluated.

**Table 2:** Computationally predicted NO mediated modifications of known auxin transport proteins (Xue et al., 2010; Liu et al., 2011). Only predictions included in the “high” threshold category are included here (10% FDR).

**Gene name**	**AGI code**	***S*-nitrosylation**		**Tyr-nitration**	
****	****	**Position**	**Peptide**	**Position**	**Peptide**
AUX 1	AT2G38120	467	LFAKCYQ**C**KPAAAAA	None predicted	
LAX 1	AT5G01240	216	MHHTKSL**C**LRALVRL	None predicted	
LAX 2	AT2G21050	None predicted		16	ETVVVGN**Y**VEMEKDG
LAX 3	AT1G77690	None predicted		14	ETVVAGN**Y**LEMEREE
PIN 1	AT1G73590	None predicted		18	MTAMVPL**Y**VAMILAY
				480	LIRNPNS**Y**SSLFGIT
PIN 2	AT5G57090	None predicted		18	LAAMVPL**Y**VAMILAY
				335	RSMSGEL**Y**NNNSVPS
				505	LIRNPNT**Y**SSLFGLA
PIN 3	AT1G70940	553	LQPKLIA**C**GNSVATF	498	LIRNPNT**Y**SSLIGLI
PIN 4	AT2G01420	525	LQPKIIA**C**GNSVATF	18	LTAVVPL**Y**VAMILAY
				470	LIRNPNT**Y**SSLIGLI
PIN 7	AT1G23080	425	NGLHKLR**C**NSTAELN	None predicted	
ABCB 1	AT2G36910	1062	ALVGPSG**C**GKSSVIS	743	MIKQIDK**Y**CYLLIGL
				908	EAKIVRL**Y**TANLEPP
				1155	LPEGYKT**Y**VGERGVQ
				1256	KNHPDGI**Y**ARMIQLQ
ABCB 4	AT2G47000	918	IRTVASF**C**AEDKVMN	277	NKHLVTA**Y**KAGVIEG
		1208	QEALDQA**C**SGRTSIV		
ABCB 19	AT3G28860	98	VYLGLVV**C**FSSYAEI	240	QVRTVYS**Y**VGESKAL
		1215	STIRGVD**C**IGVIQDG	595	LIAKSGA**Y**ASLIRFQ
TIR1	AT3G62980	516	RSLWMSS**C**SVSFGAC	450	LTDKVFE**Y**IGTYAKK
		551	PDSRPES**C**PVERVFI		

*The position of the cysteine or tyrosine amino acid predicted to be modified is give and indicated in bold.*

## CONCLUSION AND FUTURE DIRECTIONS

There is increasing evidence to support a role for NO-mediated PTMs of proteins in the regulation of plant cellular processes by eATP. Although numerous plant proteins have been predicted to undergo these changes and experimentally shown to have them in various conditions, the regulatory role of these PTMs remains to be demonstrated in all but a few cases. Given the central role of auxin in plant growth control, it is likely that the dramatic effects of extracellular nucleotides on auxin transport account for many of their effects on plant growth. Thus, a more complete understanding of how NO regulates auxin transport, whether by PTM of auxin transporters or by other mechanisms, will be key to clarifying why eATP-induced NO production is a necessary step in transducing extracellular nucleotide effects on plant growth and development.

## Conflict of Interest Statement

The authors declare that the research was conducted in the absence of any commercial or financial relationships that could be construed as a potential conflict of interest.
